# Progress in Improving Low-Cost Measurement of Feeding Behaviors and Diets^☆^

**DOI:** 10.1016/j.cdnut.2024.104503

**Published:** 2024-11-02

**Authors:** Mary Arimond, Valerie L Flax

**Affiliations:** 1 Independent Consultant, Takoma Park, MD, United States; 2 RTI International, Research Triangle Park, NC, United States

**Keywords:** nutrition surveys, infant, women, data accuracy, bias, validity

## Abstract

Information on infant and young child feeding practices and on the diets of vulnerable population groups is needed for global and national monitoring and for evaluating the impacts of interventions. Several population-level indicators have been developed to meet this need, but research to support selection of best data collection practices has been lacking. This supplement brings together a set of recent studies that address measurement issues with the objective of advancing knowledge of best practices, with a focus on low-cost measurement methods. Topics include social desirability bias, recall bias, and the cognitive validity of specific approaches and wording for asking questions on diets. The studies in the supplement provide examples of methods and results that can improve measurement and also identify gaps and areas where additional work is required.

## Introduction

Globally, undernutrition and poor diet quality are among the most important drivers of poor health [1,2], yet high-quality diets remain out of reach for far too many. In 2021, UNICEF reported that nearly half of infants and young children (6–23 mo) consumed no fruits or vegetables and more than half consumed no meat, fish, or eggs on the previous day [3]. In 2021, 3.2 billion people, or ∼40% of the global population, could not afford a healthy diet [4].

Information on feeding practices and diet quality is needed for a range of purposes, including for global and national population-level monitoring and for evaluating the impacts of programs and other interventions [[5], [6], [7]]. Individual-level data on feeding practices and diets are also needed in studies seeking to establish or further elucidate relationships to outcomes, such as cognitive development, growth, morbidity, and mortality. Acceptable levels of precision in measurement depend on the context and uses of the data, and some methods and indicators are acceptable for population-level monitoring but not for use at individual level.

For infants and young children, WHO and UNICEF defined population-level indicators to track breastfeeding practices in 1991 [8] and added a set of complementary feeding indicators in 2008; the latter included measures of feeding frequency and food group diversity [9]. Recently, these have been updated and expanded to include indicators of consumption of unhealthy foods and beverages [10]. The indicators were primarily designed for global and national monitoring but have found wide use in programs. They were designed to be relatively of low cost and feasible to collect in large multitopic surveys.

A similar low-cost indicator of food group diversity was developed for women of reproductive age as a proxy for micronutrient adequacy [11], but broader indicators of diet quality that include elements related to chronic disease risk reduction and that cover additional population groups have been lacking. To fill this gap, several diet quality indicators have recently been proposed for global use across population groups [12,13].

For the feeding and diet indicators described above, there are challenges and unanswered methodological questions related to measurement. Social desirability and recall biases are challenging in all surveys using questions based on recall, including surveys with questions on diets [14]. Social desirability bias may be larger when measurement follows an intervention [15,16]. In addition, specific methodologic choices (eg, preambles to food group recalls, question order, wording of questions, or use of prompts or visual aids) can impact survey results and indicator estimates, yet research to support selection of best data collection practices has been lacking.

This supplement brings together a set of recent studies from a variety of geographic settings and with overlapping aims, each of which addresses 1 or more measurement issues related to infant and young child feeding and diets. Taken together, the studies highlight recent progress on longstanding measurement issues while identifying remaining knowledge gaps.

Longstanding measurement issues include the following:

• How to identify social desirability biases in responses;
• Choice of recall method in surveys aiming to capture food group consumption; specifically, both list-based and “open” recalls (similar to the first pass in a multiple-pass 24-h recall) have been used [17];
• Selection of sentinel foods to include in food item lists for each food group [10]; and
• Specifics of food group questions, including the text used to introduce the recall, the order of questions, and the use of closed lists of sentinel foods compared to more open-ended lists (including wording such as “….or other vegetables”).

The supplement has a primary focus on lower cost methodologies amenable to use in large-scale multitopic surveys and at all country income levels. Most of the studies focus on infants and young children and/or women of reproductive age, but several are relevant for the general population. Most focus on measurement of population-level indicators for use in monitoring global and national trends. However, as noted, many of the same indicators have also been used in programmatic contexts and for evaluation.

## Specific Issues Addressed by Articles in the Supplement

### Social desirability

Four studies explored the impact of social desirability on survey responses. In a secondary analysis of data from an intervention trial in Kenya, Stewart et al. [18] demonstrated a strong impact of social desirability on long-term recall of breastfeeding behaviors, including early initiation and duration of exclusive breastfeeding. Note that the measurement method for exclusive breastfeeding in the study differed from that of the WHO/UNICEF indicator, which is a population-level indicator based on a recall of the previous day. Yuen-Esco et al. [19] found some evidence of an association between social desirability bias and caregiver reports of young child consumption of salty/fried foods in Cambodia. Namaste et al. [20] and Khadka et al. [21] reported results from qualitative interviews in Uganda and Nepal, which suggest that social desirability may also be a factor in women’s responses on feeding soda to children and/or consuming it themselves. Consistent with Stewart et al. [18], Namaste et al [20] also found a potential impact of social desirability on reports of early breastfeeding practices.

### Recall bias

Two studies explored the potential impact of methodologic choices on recall bias. Vanderkooy et al. [22] reported on the use of pictorial aids in multiple-pass quantitative 24-h recalls in Senegal and Nepal. Their study is of particular importance because the 24-h recall methodology they used [23] was developed for low-income and middle-income country (LMIC) settings and has been widely used and adapted [[24], [25], [26]], yet the impact of the pictorial aids had not been previously tested. Vanderkooy et al. [22] provided the first evidence of the importance of this element of the method for capturing complete recall information on healthy and unhealthy snacks. Yuen-Esco et al. [19] compared 2 methodologies for eliciting information on consumption of sentinel unhealthy foods, including sweet foods and salty/fried foods, as identified by WHO and UNICEF in the revised guide to infant and young child feeding (IYCF) indicators [10]. They compared a list-based approach, in which respondent caregivers are asked whether the child consumed specific items yesterday, to an “open” 24-h recall in which the respondent caregiver recalls all foods consumed by the child but is not prompted for specific items. They found that the list-based approach resulted in higher estimates of prevalence of consumption of unhealthy items.

The results of these 2 studies are consistent with previous work showing that snacks may be poorly recalled [27]. The studies are of high relevance given the new IYCF indicator for unhealthy food consumption and that snacks for young children are often unhealthy.

### Cognitive validity

Three studies explored the cognitive validity of specific formulations of list-based food group recall questions with a 24-h recall period. In list-based food group recalls, the enumerator reads a list of food items (or types of food), and the respondent indicates whether they consumed any of the items.

Herforth et al. [28] conducted cognitive interviews as part of the development process of a new survey tool, the Diet Quality Questionnaire (DQQ), available at: https://www.dietquality.org/. The tool is intended to meet the need for low-burden, low-cost measurement of food group consumption and to allow calculation of associated population-level indicators of diet quality. The cognitive interviews explored the impact of open-ended food list questions, which included phrases such as “any other vegetables,” compared with closed lists of specific sentinel items. The authors documented high levels of misclassification into food groups when open-ended wording was used.

In the same paper, Herforth et al. [28] also reported results from a nationally representative pilot study in Brazil, in which 2 methods were used to elicit responses about several food groups. Despite the documented issue of misclassification, the 2 methods - open-ended lists and closed lists of sentinel foods - resulted in similar estimates of prevalence of intake of the food groups at population level.

Namaste et al. [20] conducted cognitive interviews as part of a Demographic and Health Survey (DHS) pilot in Uganda, designed to mimic a multitopic population survey environment. Khadka et al. [21] conducted cognitive interviews in Nepal, using the same methodology as in the Uganda study. Results from both studies revealed specific problems with questions on milk and yogurt and gave evidence of misclassification issues for certain other food groups. Like Herforth et al. [28], the studies in Uganda and Nepal demonstrated that open-ended phrases like “or other vegetables” resulted in misclassification. However, use of closed lists resulted in the exclusion of some appropriate items. Insights gained can be used to improve questionnaires in these settings.

An additional article from Herforth et al. [29] described the adaptation of the DQQ for use in 140 countries, using a systematic key informant process to select the sentinel items representing each food group. The use of sentinel foods was previously assessed in China [30] and is currently being assessed in >30 additional countries to confirm that the selected sentinel foods accurately capture the percent of respondents consuming each food group (Anna Herforth, personal communication, 18 September, 2023).

## Discussion: Consensus Points and Knowledge Gaps

### Social desirability

Social desirability bias is likely to be present in surveys providing data for IYCF and diet quality indicators and is particularly problematic in the context of evaluations of interventions. While bias cannot be eliminated, standard survey best practices have been developed to minimize the issue and should be carefully implemented [31,32]. These include design of neutral survey questions, careful selection of enumerators, and enumerator training that covers strategies for ensuring privacy, conveying respect, building rapport, probing in a neutral manner, and striving for neutrality of verbal and nonverbal reactions to responses.

Assessing social desirability—usually measured through inclusion of a scale in surveys—can aid in interpretation and/or statistical adjustment of results. However, to our knowledge, the tools most widely used to date [[33], [34], [35]] were originally developed for use in the United States, and they may not be valid in all contexts [[36], [37], [38]]. There is a need for development of tools for measuring social desirability in a variety of LMIC contexts. If tools developed and validated in the United States are used, they themselves need to be cognitively tested or otherwise assessed for construct validity, reliability, and other characteristics. This has been done in a few LMICs [39,40] but would need to be carried out in wider variety of contexts, including for short versions of the scales.

As demonstrated by Stewart et al. [18], in the context of behavior change interventions, reporting of certain behaviors may be particularly susceptible to social desirability bias. When feasible, and particularly in the case of initial studies to establish the efficacy and effectiveness of intervention approaches, objective measures may be required [41]. In the case of exclusive breastfeeding, stable isotope techniques are available [42], although the period of measurement (14 d) aligns neither with the desired behavior (exclusive breastfeeding from birth) nor with the single-day recall used in global monitoring.

For other feeding behaviors and for diets more broadly, objective measures include unobtrusive direct observation and a limited suite of biomarkers. However, the presence of an observer can change behavior, and additional biomarkers are needed, along with strategies to make their collection more feasible and cost-effective [15]. There are also recent innovations in the use of photographic food records to estimate intakes, with validations for some population groups in some settings [43,44], but further work is needed, particularly in LMIC settings. In the meantime, in the context of program evaluations that use recall methods, plausibility is enhanced when researchers think through program impact pathways and look at coherence in indicators measured across all of these [45].

In population-level monitoring, objective, non–survey-based measures of feeding behaviors and diets are generally infeasible. Social desirability is likely to be less pronounced in monitoring than in evaluation of interventions that are promoting a particular dietary change, and in addition, bias is less problematic, so long as it is consistent across time (and space, if spatial comparisons are made). Despite the social desirability of reporting recommended practices, global IYCF indicators show that half or more of caregivers do not report positive practices—for example, for exclusive breastfeeding [46] or for feeding nutrient-rich foods [3]. It is important to consider the impacts of all types of measurement error (including bias) when interpreting results, but even imperfect indicators can have high value for population-level tracking and advocacy.

### List-based compared with open recalls for food group–based indicators

Currently, 2 different approaches to capturing food group consumption are used in major survey research programs that contribute data for global monitoring. The UNICEF Multiple-Indicator Cluster Surveys (MICS) use an open recall, and both the DHS and the Gallup World Poll use list-based approaches. The open recall is similar to the first pass of a quantitative 24-h recall. Respondents are guided through a recall of the previous day and asked to report all foods and beverages consumed by the respondents or their young children. In some cases, including in MICS, this open approach is followed by a list to ensure no items are omitted [10]. Food items reported are then categorized into food groups.

To our knowledge, there is no research to date documenting the impact of the practice of following the open recall with list-based probing for consumption of each food group not mentioned by the caregiver. This practice, intended to ensure completeness, has the potential to elicit biased responses, particularly for socially desirable foods.

In a list-based approach, the enumerator reads a list of food items or types previously organized into groups (eg, lists of sentinel items that fall into the group “dark green leafy vegetables”) and asks the respondent to indicate if they consumed any item in the group. There are advantages and disadvantages to each approach [17].

To date, there is limited and mixed evidence from comparisons of the 2 approaches. In a 3-country study (Cambodia, Ethiopia, and Zambia) of women of reproductive age, Hanley-Cook et al. [47] demonstrated moderate agreement between the 2 recall methods and an overestimation of intakes by both methods compared with weighed food records, considered a gold standard. Nguyen et al. [48] showed that the 2 approaches performed similarly for calculating minimum dietary diversity for women in 2 countries in South Asia.

Hackl et al. [49] compared both approaches to direct observation, another gold standard, but did not directly test for differences between the 2 approaches. Their 2-country study (Cambodia and Zambia) assessed equivalence in estimation of food group intake (yes or no) and minimum dietary diversity (MDD) for infants and young children. MDD estimates from both methods were equivalent to observations in Cambodia, but neither was equivalent in Zambia. At population level, the list-based estimate of MDD was closer to the observed level in both countries, and the list-based method was also less costly to implement.

In this supplement, Yuen-Esco et al. [19] provide suggestive evidence of superiority of the list-based approach for capturing consumption of unhealthy snacks by young children in Cambodia; however, this study did not include comparison to a gold standard method. Nevertheless, because they showed both a larger impact of social desirability and higher reporting of consumption with the list-based approach, their results lend plausibility to the idea that the list-based approach is superior for caregiver recall of unhealthy food consumption by young children.

It would be valuable to extend this small body of evidence with similar studies in additional urban and rural settings and global regions, using consistent methodologies. Such studies could potentially use the DQQ for the list-based arm of the study and would ideally report suites of indicators for various population groups, including newer indicators, to improve our understanding of which indicator estimates are sensitive to this methodologic choice. Given the importance of MICS and DHS data in global monitoring and the potential for differing results based on methods, this additional research is warranted.

### Cognitive validity and accuracy of list-based food group recalls

Incorporation of cognitive interviewing and/or other qualitative methodologies during questionnaire development is considered a best practice, particularly in the context of comparative survey research across cultures or countries [50]. Studies in this supplement underscore the value of cognitive interviewing in refinement of both the questions and the food item lists in food group recall survey tools.

However, the high capacity and resource requirements for undertaking this type of qualitative work may render it infeasible in many contexts. Further, sample sizes for cognitive interviewing are generally small, yet cognitive interviews that cover only a narrow subset of survey respondents—that is, which do not capture the full range of regions, cultures, or socioeconomic or literacy levels among the planned survey respondents—may yield incomplete or misleading results.

Cognitive interviewing and/or other qualitative approaches are most critical during initial questionnaire design and adaptation. In the case of the DQQ, cognitive interviews were used during the development of the tool, and this was followed by a systematic, intensive, and extensive country-level adaptation process involving around 1000 key informant interviews across 140 countries. This has resulted in questionnaires that are freely available, as a public good. The questionnaires should be usable at the national level without further adaptation in contexts where a simple, low-cost list-based questionnaire is appropriate and needed. Further adaptation might be needed when surveys focus on smaller administrative units (districts, cities) or on specific subpopulations, as the most salient sentinel foods may differ. Herforth et al. [29] outline an approach for extending the DQQ in these situations.

Areas for future work on the DQQ include ensuring that branded products mentioned on food lists are updated as needed [21]. When specific food items are promoted, as in certain agriculture-nutrition projects, these can and should be captured with separate questions.

### Conclusions

The studies in this supplement address a series of overlapping methodological questions, all related to the accuracy and precision with which feeding practices and food group consumption can be measured. Compared with research contexts, surveys for population-level monitoring may have lower requirements for precision, and some amount of random error in measurement is both inevitable and tolerable. However, it is still important to improve precision where possible, particularly when tracking trends over short spans of years. Similarly, when indicators are needed for pre–post surveys in programmatic contexts, lack of precision is a problem because true impacts can be obscured.

The studies in this supplement provide specific examples of methods and results that can improve measurement of IYCF and diet indicators. In addition, the supplement reports on the adaptation of a new tool for global use in tracking food group consumption and associated indices of diet quality. The work already undertaken means that users can now measure more consistently and accurately, with little or no additional adaptation.

However, as always, many questions remain unanswered. At present, 2 major survey programs (DHS and MICS) provide data for global monitoring of IYCF indicators using different approaches. Some of the research to date is reassuring, in showing reasonable comparability between list-based and open recall methods, but other results indicate differences. For major methodologic decisions, the burden of proof before recommending a single best practice is high, so we recommend additional work comparing the 2 approaches. Considering the list-based approach, we also recommend additional work to confirm that the DQQ sentinel food lists that have been developed accurately capture population patterns in food group consumption; some of this work is underway.

Finally, the impact of social desirability bias remains a vexing challenge in many contexts, and particularly in the context of evaluating behavior change interventions. Along with careful implementation of survey best practices to minimize bias, we also recommend additional research into development and adaptation of appropriate tools and scales for measuring social desirability bias, including short versions of scales, in a wide range of cultural contexts. When such scales are available, they can aid in interpretation and allow for adjustment of results.

We, along with all authors of this supplement, hope that the research reported will stimulate interest and momentum for additional methodologic work to improve measurement of feeding and diets, in particular in those settings where simple, low-cost methods are essential.

## Author contributions

The authors’ responsibilities were as follows—MA, VLF: conceptualized the paper; MA: wrote the paper; VLF: commented substantively on all drafts; and both authors: approved the final draft.

## Data availability

This article is an introduction to a journal supplement and does not present results of primary data collection or secondary data analysis.

## Funding

The preparation of this paper was supported, in whole or in part, by the Bill & Melinda Gates Foundation (OPP1190179) and by the United States Agency for International Development (USAID) and the Bill & Melinda Gates Foundation through The DHS Program (#720-OAA-18C-00083 and INV-008034). Under the grant conditions of the Foundation, a Creative Commons Attribution 4.0 Generic License has already been assigned to the Author Accepted Manuscript version that might arise from this submission.

## Conflict of interest

The authors report no conflicts of interest.
